# Differences between Han Chinese and Caucasians in transcranial magnetic stimulation parameters

**DOI:** 10.1007/s00221-013-3763-2

**Published:** 2013-11-15

**Authors:** Xiang Yi, Karen M. Fisher, Ming Lai, Kashif Mansoor, R. Bicker, Stuart N. Baker

**Affiliations:** 1 School of Mechanical and System Engineering, Newcastle University, Newcastle upon Tyne, NE1 7RU UK; 2 Institute of Neuroscience, Medical School, Newcastle University, Framlington Place, Newcastle upon Tyne, NE2 4HH UK; 3 Department of Clinical Neurophysiology, Royal Victoria Infirmary, Newcastle upon Tyne, NE4 1LP UK

**Keywords:** Han Chinese, Caucasians, TMS parameters, Different

## Abstract

The study was conducted to investigate the difference between Han Chinese and Caucasians on various parameters measured from responses to transcranial magnetic brain stimulation (TMS). Sixteen subjects were studied in each group. A circular coil at the vertex was used for stimulation, whilst recording surface electromyograms from right first dorsal interosseous. In the passive state, motor-evoked potential (MEP) threshold, MEP recruitment, short-interval intracortical inhibition (SICI) and intracortical facilitation were measured. The MEP threshold, recruitment and silent period were also measured in the active state. Chinese subjects showed significantly higher passive thresholds (*P* < 0.005), less inhibition of the motor response (SICI, *P* < 0.0005) and a shorter silent period (*P* < 0.05). Differences in SICI appeared to be a consequence of the differences in passive threshold and were not seen when active threshold was used to determine the conditioning stimulus intensity. Differences in silent period may also reflect differences in cortical excitability rather than inhibitory processes, as they were not seen when the silent-period duration was expressed as a function of MEP size, rather than TMS intensity. There appears to be a significant difference in some TMS parameters between Han Chinese and Caucasian subjects. This may reflect an underlying difference in cortical excitability.

## Introduction

Transcranial magnetic brain stimulation (TMS) is an important neurophysiological method. Measurement of the threshold stimulus intensity required to elicit a contralateral muscle response can yield information on the integrity of corticospinal pathways. In addition, using paired-pulse paradigms (Kujirai et al. 1993; Nakamura et al. 1997) or by measurement of the silent period (Ziemann et al. 1993), TMS can probe the function of local cortical circuits. As well as extensive use in the basic sciences, TMS has found a useful role in diagnosis of a wide range of diseases (Chen et al. 2008). Such use relies on detecting abnormal measurements in patient populations compared to normative control data. We were intrigued by anecdotal observations within our own group, confirmed by conversations with other centres that healthy Chinese subjects often have high TMS thresholds compared with other races. If true, this would have obvious and important implications for the diagnostic use of TMS. Accordingly, we conducted a study to compare various TMS parameters between Chinese and Caucasian racial groups in a sample of young healthy volunteer subjects. We show that there are indeed significant differences, which should be taken into account when interpreting the results of investigations in individual patients.

## Methods

Thirty-two healthy Han Chinese and white European volunteers (16 in each racial group, eight males and eight females, age 21–28; all right handed except for a single male European subject who was left handed by self-report) consented to participate in this study, which was approved by the Local Research Ethics Committee of Newcastle University Medical School. The majority of subjects (30/32) were recruited from outside of the research group so as to reduce any potential bias in our samples. Only two subjects who were laboratory members (one Caucasian, one Chinese) had previous experience of TMS; in both of these cases, the experimenter was blind to their previous results.

Surface electromyogram (EMG) signals were recorded (band pass 30 Hz to 2 kHz) from the right first dorsal intercosseous (1DI) using bipolar surface electrodes. For active state measurements, subjects maintained a steady contraction of 1DI at a level of 5 % of their maximum voluntary contraction (MVC); this was ensured via visual feedback of the mean-rectified EMG using a computer display.

Two Magstim 200 stimulators (The Magstim Company Ltd, Dyfed, UK) connected via a Bistim unit were used for the motor cortical stimulation along with a 13-cm-outside-diameter circular coil. Stimulus intensities are expressed as a percentage of the maximum stimulator output (MSO). The coil was placed over the vertex, with current direction optimal for left hemisphere activation.

The passive threshold was initially identified by viewing the stimulus-triggered EMG on an oscilloscope screen. TMS intensity was increased by 5 % increments until a reproducible MEP was observed. After this, the stimulus intensity was changed in 1 % steps until threshold was located (defined as a visible response to 5/10 stimuli). We refer to this value as the ‘online threshold’.

Three experiments were carried out. In the first experiment, subjects sat at rest whilst single-pulse TMS was delivered at intensities ranging from 15 % below the online threshold up to a level where the MEP saturated, in 5 % steps. Ten stimuli were delivered at each intensity.

The second experiment used paired-pulse stimulation over motor cortex, also with the subject at rest. A subthreshold stimulus (0.8× online threshold) preceded a suprathreshold stimulus (1.2× online threshold) with intervals of 3 ms (short-interval intracortical inhibition, SICI) or 10 ms (intracortical facilitation, ICF). The different intervals, together with a control condition of a single suprathreshold stimulus, were randomly interleaved (0.2 Hz; approximately 20 stimuli per condition).

In the third experiment, subjects made a contraction of 1DI at 5 % MVC and responses were recorded to an ascending sequence of TMS intensities, from 20 % below online threshold until the MEP was saturated, in 5 % steps. Ten stimuli were given at each intensity.

A second series of ten Chinese and nine Caucasian subjects participated in an experiment to measure SICI at different intensities of conditioning stimulus. In these subjects, we first determined accurate active and passive thresholds by recording a series of responses to stimuli with different intensities given in randomized order; 5–6 intensities were tested around the approximate threshold, separated by 1 or 2 % increments. Threshold was defined as the first intensity with a response that was significantly different from baseline using a two sample *t* test. Recordings of SICI were then made, with the conditioning intensity set to 0.5, 0.6, 0.7, 0.8, 0.9, 1.0 and 1.1× passive threshold; different intensities were randomly interleaved. Ten repetitions of each conditioning intensity were given. A second recording then repeated the measurement of SICI, but using conditioning intensities of 0.5, 0.6, 0.7, 0.8, 0.9, 1.0, 1.1 and 1.2× active threshold.

Stimulus markers and EMG waveform were captured continuously to a computer (5 kHz sampling rate; Spike 2 software and 1401 intelligent laboratory interface, CED Ltd, Cambridge, UK). Offline analysis separated responses according to condition and compiled averages of rectified EMG. Single sweep responses were measured as the area of the rectified EMG between the MEP onset and offset, judged from the averaged response; single-subject plots show the mean ± SEM. A sigmoid curve was used to fit the relationship between response amplitude and intensity for both passive and active MEP recruitment relationships, according to the following relation:

$$R = R_{\rm{max} } \frac{1}{{1 + {\text{e}}^{{\frac{{I_{50} - I}}{k}}} }}$$

where *R* is the response amplitude at intensity *I*. From the parameters of this curve, we measured the intensity at which the response was half maximal (*I*_50_), the maximal response (*R*_max_) and the parameter *k*, which is related to how quickly the response rises (the slope at intensity *I* = *I*_50_ is d*I*/d*R* = *R*_max_/4*k*). Passive and active thresholds were estimated as the first intensity where the response was significantly different from zero; this is denoted here the ‘offline threshold’. In the active state, the silent-period duration was estimated by measuring the time at which the average returned to the baseline level. The level of background contraction has been shown to have varied effects on the duration of the silent period (Taylor et al. 1997; Wilson et al. 1993), therefore subjects were required to maintain a steady contraction at 5 % MVC throughout. This was achieved by providing visual feedback of the rectified and smoothed EMG level on a computer screen. Responses to paired-pulse stimulation at a given interval (3 ms and 10 ms) were normalized as a percentage of the response (measured as area of the rectified EMG, as described above) to the suprathreshold stimulus alone to give values for SICI and ICF. Box plots were compiled to summarize all parameters in each racial group; significant differences were assessed using the Mann–Whitney *U* test.

## Results

**Fig. 1:**
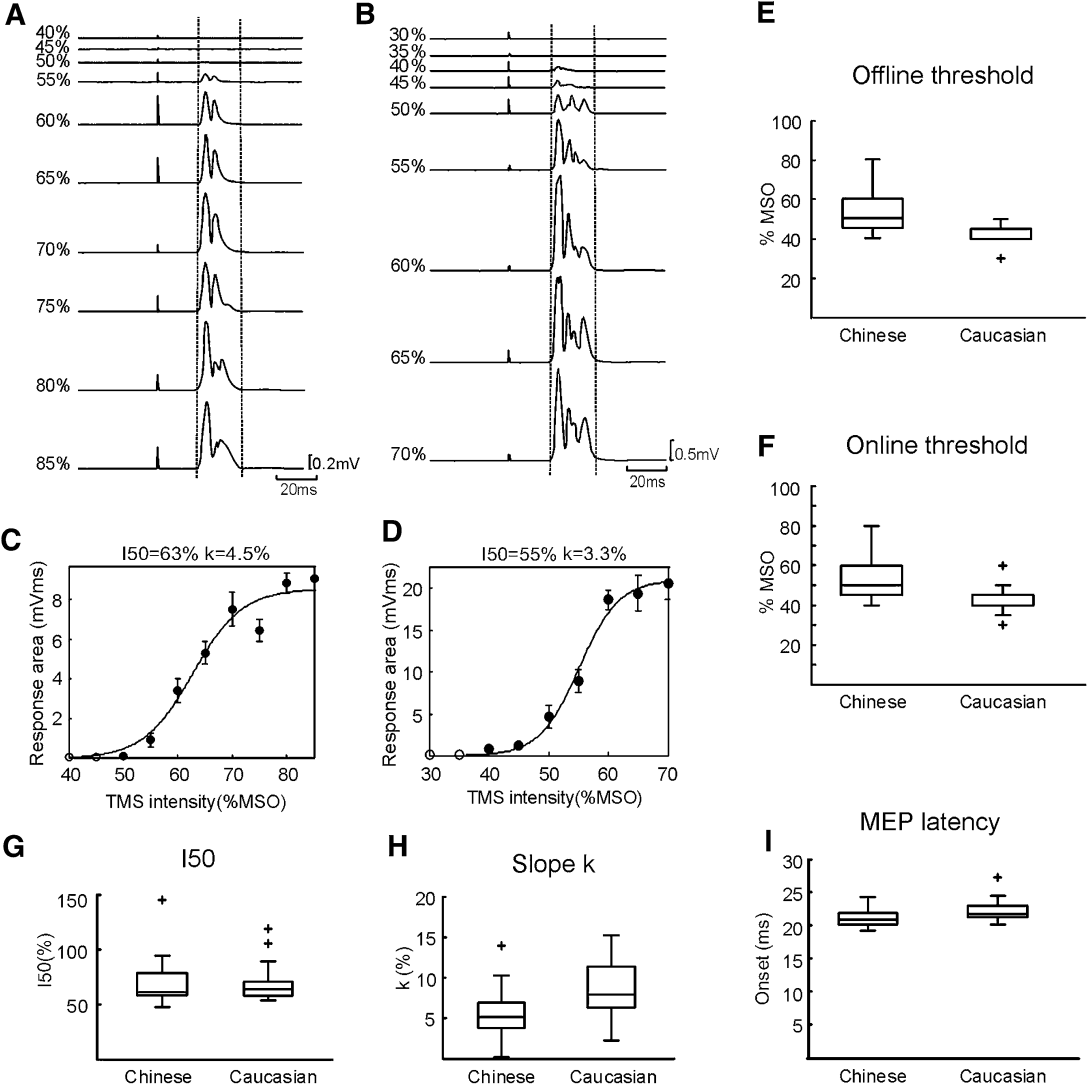
Results of single-pulse stimulation in the passive state. Averaged rectified EMG from 1DI and MEP recruitments for a Chinese (**a**, **c**) and Caucasian (**b**, **d**) subject following single-pulse stimulation at different intensities. *Vertical dashed lines* show the response region.* Filled circles* show responses significantly different from zero (*t* test, *P* < 0.05). *Error bars* show standard errors. **e**–**i** *Box plots* showing results across the subject population for offline threshold, online threshold, *I*_50_ (the stimulus intensity producing a half-maximal response), parameter *k* (indicating how quickly responses grew with increased intensity) and MEP latency. MSO, maximum stimulator output

Figure 1a–d shows results from two individual subjects. Figure 1a shows averaged EMG responses following single-pulse TMS at different intensities in a Chinese subject; these were quantified to yield a MEP recruitment curve in Fig. 1c. Figure 1b, d presents similar displays for a single Caucasian subject. For these two cases, the Chinese subject had a higher offline threshold (50 %) than the Caucasian (40 %); a similar trend was seen in the *I*_50_ (63 vs 55 %). The response grew more slowly with increases in intensity in the Chinese compared with the Caucasian subject (value of parameter *k* of 4.5 and 3.3 %, respectively).

The remainder of Fig. 1 shows group data, presented as box plots for each measure made from the responses at rest. Both offline threshold (Fig. 1e; mean Chinese: 51.6 %, mean Caucasian: 41.9 %) and online threshold (Fig. 1f; mean Chinese: 55.6 %, mean Caucasian: 43.3 %) showed clear significant differences between the two groups (*P* < 0.005 and *P* < 0.0005, respectively). Parameter *k* was also found to be significantly different between groups (Fig. 1h; *P* < 0.05) By contrast, there were no significant differences in *I*_50_ (Fig. 1g, *P* > 0.05) or in the population variance (offline threshold, standard deviation; Chinese: 10.4 %, Caucasian: 7.5 %; *P* > 0.05, *F* test). There was a small, but just significant difference in MEP latency between the groups (mean 21.1-ms Chinese vs 22.2-ms Caucasian,* P* = 0.042).

**Fig. 2:**
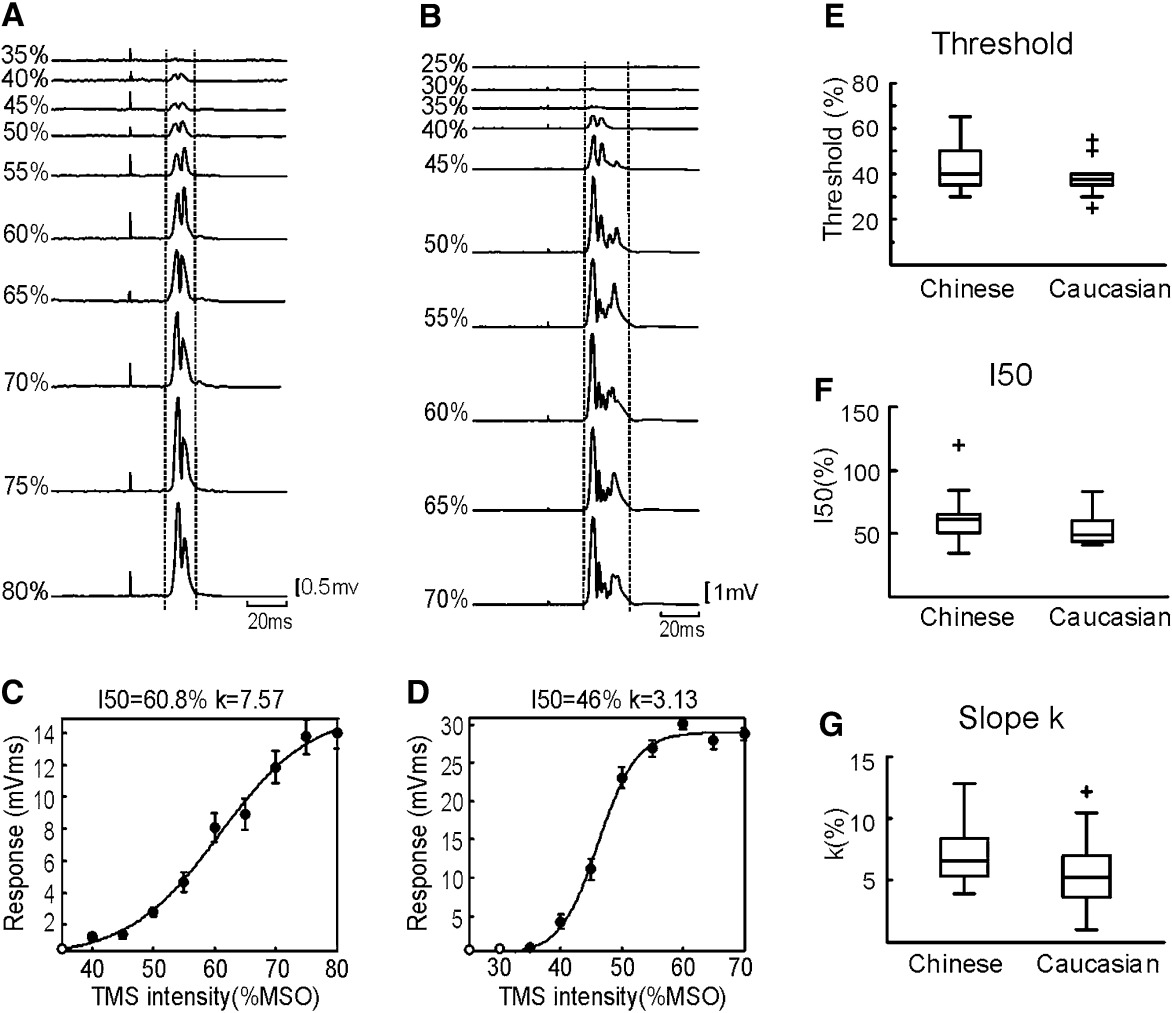
Results of single-pulse stimulation in the active state. Averaged rectified EMG from 1DI and MEP recruitments are shown from a Chinese (**a**, **c**) and a Caucasian (**b**, **d**) subject following single-pulse stimulation at different intensities. *Vertical dashed lines* show the response region.* Filled circles* show responses significantly different from zero (*t* test, *P* < 0.05). *Error bars* show standard errors. **e**–**g**, population data for active threshold, *I*_50_ and parameter *k*. MSO, maximum stimulator output

Figure 2 presents similar measurements made in the active state. Single-subject responses and the corresponding recruitment curves are shown for a Chinese subject (Fig. 2a, c) and a Caucasian (Fig. 2b, d). In these subjects, the Chinese had a higher threshold (40 %) than the Caucasian subject (35 %) and larger *k* (7.6 vs 3.1 %), indicating that responses grew more slowly with increasing intensity. Across the population, there was no significant difference in active threshold between the two populations (Chinese: 42.8 %; Caucasian: 38.1 %; Fig. 2e, *P* > 0.05). In the active state, neither the *I*_50_ nor the *k* parameter differed significantly between Chinese and Caucasians (Fig. 2f, g, *P* > 0.05). There was also no significant difference in the population variance (offline threshold, standard deviation; Chinese: 9.8 %, Caucasian: 7.0 %; *P* > 0.05, *F* test).

We were concerned that our finding of significant differences in the passive threshold, but not active threshold, might simply be an effect of statistical thresholding—in other words, that similar differences might actually exist, but an effect was not detected for active threshold simply due to chance fluctuations across the population. To address this, we calculated for each subject the difference between passive and active threshold. This difference was significantly greater for Chinese than for Caucasian subjects (mean difference 8.8 vs 3.8 %, *P* < 0.01). This indicates that there is a genuine difference in the results for active and passive threshold and that this is not simply a statistical artefact.

**Fig. 3:**
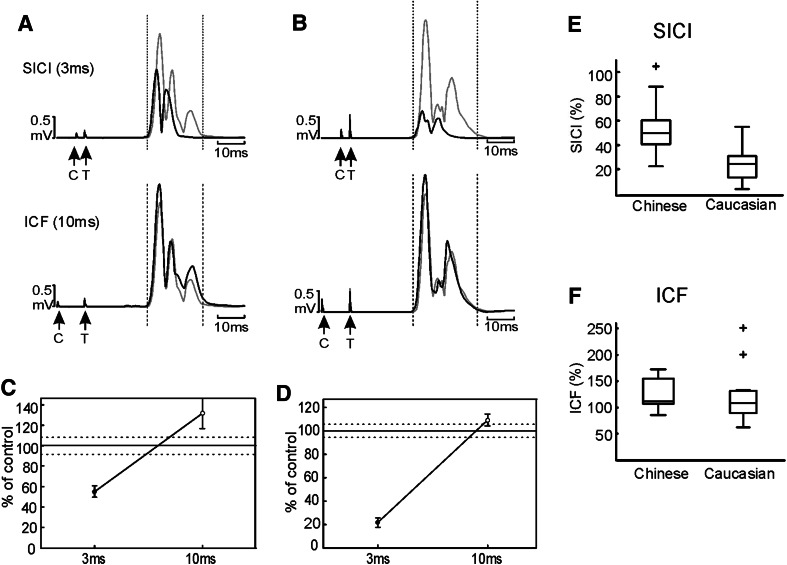
Results of paired-pulse stimulation in the passive state. Averaged rectified EMG from 1DI is shown from a Chinese (**a**) and a Caucasian (**b**) subject following paired-pulse stimulation at the interval of 3 ms (SICI) and 10 ms (ICF). *Vertical dotted lines* show response region. *Black traces* indicate the conditioned response, and *grey lines* the control response. ‘C’ indicates the conditioning stimulus, and ‘T’ the test stimulus. **c**, **d** Response area as a percentage of control for the subjects shown in (**a**, **b**). The *solid horizontal lines* show 100 %; *dotted lines* indicate standard error of the mean (SEM) of the control response. *Filled markers* indicate responses significantly different from control (*t* test, *P* < 0.05). The *error bars* are SEM of the conditioned responses. **e**, **f** *Box plots* showing population data on SICI and ICF

Figure 3 presents data on the SICI/ICF measures of intracortical circuitry from 15 Chinese subjects and 15 Caucasians; two subjects were excluded because of technical problems with the recordings. Example traces are shown from a Chinese and Caucasian subject in Fig. 3a, b, respectively. In each case, the grey trace shows the response to the test pulse alone; this is overlain with the conditioned response (black trace). The response was suppressed at the 3 ms inter-stimulus interval (SICI) and facilitated at 10 ms (ICF). Figure 3c, d presents quantification of the facilitation or suppression for these subjects, whilst the results across the population of subjects are shown in Fig. 3e, f. For the SICI paradigm, responses were more suppressed for the Caucasian compared with the Chinese subjects (Fig. 3e, *P* < 0.0005), whilst no significant differences were seen in ICF (Fig. 3f, *P* > 0.05).

**Fig. 4:**
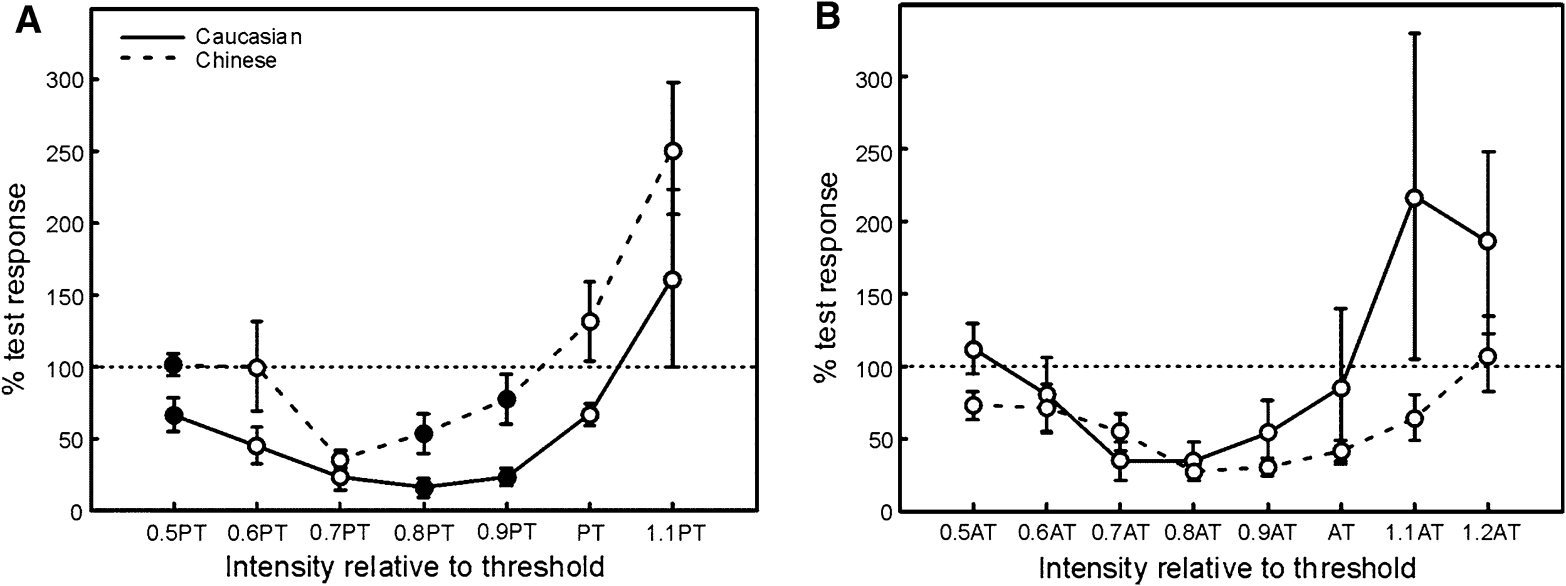
Dependence of SICI on conditioning stimulus intensity. Intensity was determined relative to passive motor threshold in (**a**) and relative to active motor threshold in (**b**). *Filled markers* indicate responses significantly different between the two racial groups (*t* test, *P* < 0.05). The *error bars* are SEM of the conditioned responses. Results have been averaged over subjects in each racial group and come from a separate series of experiments, in different subjects, compared with those in the other figures of this paper

One possibly confounding factor in the paired-pulse experiments is that the intensity of the conditioning stimulus was determined relative to the passive threshold, which is often the approach used in measurement of SICI. However, we have shown that the passive threshold was higher in Chinese compared with Caucasian subjects (Fig. 1e, f), whilst the active threshold was similar. The effective intensity of the conditioning stimulus may thus have been higher for the Chinese subjects. This could influence measurement of SICI, which is known to be sensitive to the intensity of the conditioning stimulus. In order to investigate this possibility, we conducted a second series of experiments that measured SICI at different conditioning stimulus intensities. In the first of these recordings, we used conditioning stimuli set at various fractions of the passive threshold (Fig. 4a). As in the results presented above, there were differences in SICI between the two racial groups; these reached statistical significance at intensities of 0.5, 0.8 and 0.9× passive threshold. A second recording repeated measurement of SICI, but used intensities of conditioning stimulus determined relative to the active motor threshold. As shown in Fig. 4b, this revealed no significant differences in SICI between the racial groups. These data thus confirm that the measured differences in SICI seen in Fig. 3e are likely to result directly from the differences in passive threshold illustrated in Fig. 1e, f, and its use to determine conditioning stimulus intensity.

**Fig. 5:**
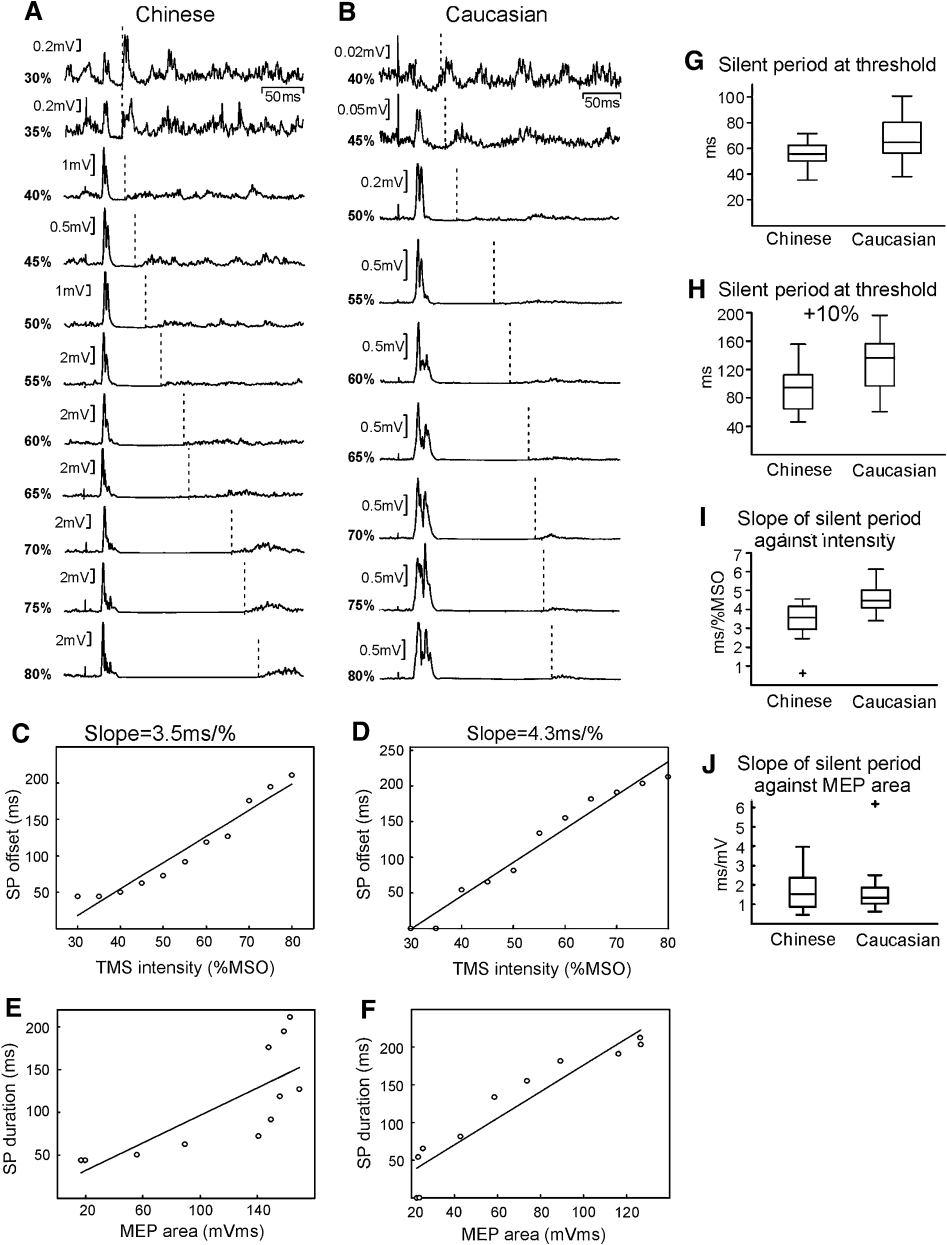
Results of silent-period measurement. Averaged rectified EMG from 1DI is shown from a Chinese (**a**) and a Caucasian (**b**) in the active state. The *vertical dotted lines* indicate the silent-period offset at different intensities. Zero levels of averages are not shown for clarity and do not necessarily align between traces in (**a**) and (**b**). **c**, **d** Relationship between silent-period offset and intensity, for the subjects shown in (**a**, **b**). Overlain line is the best-fit *straight line*, with slope calculated as shown above each plot. **e**, **f** Relationship between silent-period offset and MEP area, for the subjects shown in (**a**, **b**). Overlain is the best-fit *straight line*. **g**, **h** *Box plots* of population data on silent period at threshold and threshold +10 %, and the slope of the relationship between silent-period offset and intensity (**i**) and MEP size (expressed as a percentage of the maximum MEP recorded, **j**)

Figure 5 illustrates data on the silent period, measured in 15 Chinese subjects and 16 Caucasians; no silent period could be reliably seen in one subject. Individual responses from two subjects in Fig. 5a, b showed a clear silent period. The estimated offset time of the silent period has been marked by a vertical dotted line for each intensity where a silent period could be discerned. Plots of the offset latency versus intensity revealed an approximately linear relationship (Fig. 5c, d), allowing estimation of the slope of the best-fit line. At threshold and intensities 10 % MSO above threshold, the silent period was significantly longer in Caucasians (Fig. 5g, h; *P* < 0.05). The slope of the silent-period duration versus intensity relationship was also higher in Caucasians than Chinese (Fig. 5i; *P* < 0.02). One possible confounding factor is that the MEP recruitment curves may differ between the two groups. It is known that the silent period varies with MEP size (Wu et al. 2000; Orth and Rothwell 2004); the observed differences in silent period could thus merely be a consequence of different recruitment curves. To control this, we plotted the silent-period duration against the MEP size, rather than against intensity. For each subject, we again measured the slope of the best-fit line (mean *r* ^2^ was 0.84); examples for two individual subjects are shown in Fig. 5e, f (Fig. 5e, f; *r* ^2^: 0.63 and 0.89, respectively). The slope of the relationship between silent period and MEP size was not significantly different between Caucasian and Chinese subjects (Fig. 5j; *P* > 0.05).

## Discussion

Previous work showed that parameters measured from TMS responses vary widely across a healthy population, but failed to find correlation with either age or sex of subjects (Wassermann 2002). This study provides the first objective evidence that there are differences between TMS measurements across different racial groups, supporting anecdotal accounts from several laboratories. A small difference was seen in MEP latency between Chinese and Caucasian subjects. However, this result was especially influenced by a single outlying Caucasian subject with a particularly long latency, and there were no significant differences in latency if this subject was excluded. This suggests that central motor conduction time (CMCT) will not show great differences between the groups and can probably reasonably be interpreted without reference to the patient’s race. By contrast, passive threshold, SICI and silent period all appeared different. It would be unsafe to use normative data on these measures gathered from healthy volunteers in a single racial group to diagnose abnormality in a wider range of patients. Furthermore, gathering normative data in heterogeneous populations without consideration of race will increase the variability in the measures and hence reduce their sensitivity to detect abnormality.

Our results appear to show two distinct differences between Chinese and Caucasian subjects. The first is a lower passive threshold in Caucasians; by contrast, the active threshold was similar. The most obvious possible explanation for differences is that the skull shape is subtly different between the two groups. This could modify current flows within the brain, leading to a different effective stimulus at the same percentage of maximum stimulator output. However, such an explanation cannot account for the unchanged active threshold. If changes in current flow underlie our observations, we would expect a simple shifting to higher intensities of all measures in Chinese subjects, rather than a selective effect on the passive threshold. Similarly, differences in the location of the M1 hand representation relative to the vertex could not account for the observed results.

In switching from the passive to the active state, there are changes in both corticospinal (Baker et al. 1995; Di Lazzaro et al. 1998) and motoneuron excitabilities. Our results suggest that overall excitability is different between Chinese and Caucasian subjects at rest, but that these differences disappear during an active contraction. This implies that the changes from rest to active must be more profound for the Chinese subjects. It is unclear whether these differences lie at the spinal or cortical level; further studies, for example using H reflex testing or responses to corticospinal stimulation at the cervicomedullary junction (Ugawa et al. 1991), could resolve this issue.

A second apparent difference is in the extent of intracortical inhibition. Caucasians had greater inhibition of a test response by a preceding conditioning response in the SICI protocol, and a longer-duration silent period. One possible explanation is that these results are an artefact of the measurement process. The SICI protocol determined the conditioning stimulus intensity as 0.8× the passive motor threshold, estimated online—this produces maximal inhibition in healthy subjects (Kujirai et al. 1993). If cortical or spinal excitability differences affect the threshold estimate, but not the intracortical inhibition, Chinese subjects would receive a stronger stimulus than Caucasians relative to the threshold needed to produce SICI. Condition-test paradigms are potentially highly sensitive to stimulus intensity (Matthews 1999), with a U-shaped dependence on intensity previously reported (Kujirai et al. 1993). When we measured SICI at a range of conditioning intensities, we found differences between the two racial groups only when conditioning intensity was expressed relative to passive motor threshold and not relative to active motor threshold (Fig. 4). This suggests that whatever process produces the elevated passive threshold in Chinese subjects does not also influence the threshold for activation of the inhibitory intracortical elements measured by SICI. The differences in SICI thus seem to be merely an artefact of using passive threshold to set the conditioning stimulus intensity and do not reflect differences in cortical inhibitory processes.

The silent period results are also likely to be secondary to changes in the threshold or MEP recruitment. There was a clear difference in silent period between the racial groups at an intensity defined relative to active threshold (which was similar between groups) and also in the slope of the silent period versus TMS intensity. However, differences in silent-period recruitment were not seen when data were expressed as a function of MEP size rather than stimulus intensity. It is possible therefore that the observed differences in silent period are (as for SICI) produced as a side effect of differences in cortical excitability, rather than genuinely representing different cortical inhibitory processes.

In this study, the relationship between stimulus intensity and silent-period duration was well fitted by a straight line. A sigmoid curve may provide a more suitable fit (Kimiskidis et al. 2005), since silent-period duration does not increase above a certain stimulus level (Valls-Sole et al. 1994). However, in our experiments, the duration of the silent period did not reach a plateau for most subjects, justifying the approximation of a linear fit in this case.

No differences were found between the two groups on the ICF paradigm. There is considerable uncertainty on the pathways which mediate the facilitation of the test response at condition-test intervals around 10 ms, with some authors suggesting that ICF may not reflect intracortical processes at all, but rather changes in spinal excitability (Di Lazzaro et al. 2006; Chen et al. 2008; Fisher et al. 2012). It is conceivable that differences between racial groups could be exploited to investigate this issue further; any pathway which shows differences between Chinese and Caucasians is unlikely to contribute to ICF.

In conclusion, we show differences between Han Chinese and white European healthy subjects on several TMS parameters. Although the causes of these differences remain to be clarified, future clinical and experimental studies using TMS should be aware of the differences. Routine recording of the racial group of subjects would be a wise addition to experimental protocols, as this could allow improved post hoc interpretation of individual results that appear as outliers.
